# Numerical Study of Heat and Mass Transfer during Cryopreservation Process with Application of Directed Interval Arithmetic

**DOI:** 10.3390/ma14112966

**Published:** 2021-05-31

**Authors:** Alicja Piasecka-Belkhayat, Anna Skorupa

**Affiliations:** Department of Computational Mechanics and Engineering, Silesian University of Technology, Konarskiego 18A, 44-100 Gliwice, Poland; alicja.piasecka-belkhayat@polsl.pl

**Keywords:** cryopreservation, heat transfer, mass transfer, interval finite difference method, directed interval arithmetic

## Abstract

In the present paper, numerical modelling of heat and mass transfer proceeding in a two-dimensional axially symmetrical articular cartilage sample subjected to a cryopreservation process is presented. In the model under consideration, interval parameters were assumed. The heat transfer process is described using the Fourier interval equation, while the cryoprotectant transport (DMSO) across the cell membrane is analyzed using a two-parameter model taking into account the simulation of the water volume in the chondrocytes and the change in DMSO concentration over time. The liquidus tracking (LT) protocol introduced by Pegg et al. was used to model the cryopreservation process. This procedure divides the heating and cooling phases into eight and seven steps, respectively, allowing precise regulation of temperature and cryoprotectant (CPA) concentration of bathing solutions. This protocol protects chondrocytes from ice crystal, osmotic stress, and electrolyte damage. The obtained interval concentrations of cryoprotectant in chondrocytes were compared with previous simulations obtained using the deterministic model and they are mostly in agreement with the simulation data.

## 1. Introduction

Cryopreservation is the storage of cells, tissues, or other biological structure at low temperatures without injuring them. This approach has many practical applications, including usage in medicine. The process is applied to preserve stem cells (tissue engineering research) or to cryobank transported organs (in transplantology) [1,2,3].

Cryopreservation can be accomplished via two methods, slow freezing and vitrification. These techniques differ principally in cooling rate and in cryoprotectant (CPA) concentration. The slow freezing method is carried out at a rate of approx. −1 °C/min with a low CPA concentration of about 1–2 M. This procedure has a low risk of tissue damage from osmotic shock to cells or the chemical toxic effects of CPA. The disadvantage is the possibility of tissue injury caused by ice crystal formation. On the other hand, vitrification is the conversion from a liquid to a vitreous phase at a cooling rate of approx. −100 °C/min to avoid the ice crystal formation. This method uses a high concentration of CPA (about 4–8 M), which increases the risk of tissue damage through CPA toxicity and the possibility of osmotic shock to cells [2,3].

However, cells such as chondrocytes in articular cartilage are susceptible to injury due to the forming of ice crystals, which makes cryopreservation using conventional methods difficult [4]. To solve this problem, Pegg et al. [5] and Wang et al. [6] used another procedure called the liquidus tracking (LT) method. This technique was devised by Farrant (1965) [7] and further advanced by Elford et al. (1972) [8]. This approach precisely regulates the temperature and the CPA concentration of bathing solutions. The temperatures in the sample are above or on the liquidus line (the melting point is changed by the CPA impact). This prevents ice crystallization and the exposition of the cryopreserved tissues to high concentrations of CPA at the same time [4].

An opportunity to further develop cryopreservation is to create appropriate mathematical and numerical models. A multi-physical field weak coupling problem mainly consists of modelling the following processes: heat transfer, including the crystallization and moving boundary problem, as well as mass transport at the macroscale in the extracellular matrix and at the microscale—through the cell membrane [2]. Cryopreservation by slow freezing [9] and by vitrification [10,11,12] can both be analyzed. These models are considered not only for articular cartilage [12,13,14,15,16] but also for other biological tissues or cells, such as stem cells [9,17,18].

Modelling mass transfer across the cell membrane is used to estimate the CPA concentration in cells (chondrocytes for articular cartilage). The model currently applied is two-parameter (2-P) formalism [14,19,20,21,22], which is an extension of the Jacobs and Steward model (1932) [23,24]. They proposed two combined differential equations describing the transport of permeable solute and water [23,25]. Kedem and Katchalsky (1958) [26] prepared another solution to the mass transfer problem at the microscale, where the membrane permeability equation is related to three parameters [18,20,22,25,27]. In the articles [28,29,30], alternative models suggested by Mazur (1963) [31] and expanded by Levin et al. (1976) are presented [32].

The microscale mass transfer model should be supplemented by the following analyses: mass transport in the extracellular matrix and thermal processing in the tissue. The model of mass transport at the macroscale is based on Fick’s second law, which provides for a concentration at a given time associated with diffusion [10,12,13,14,15,16,33]. The temperature distribution is determined from the Fourier equation [9,10,11,13,14]. In the modelling of thermal processes, the phase changes [34,35,36] or the crystallization [2,9,10,11] phenomena, as well as the moving boundary problem [2,36,37], can be considered.

Mathematical models of cryopreservation in previous works determined the parameters of the equations as deterministic values. As a consequence, the thermal processes and mass processes can be inaccurately interpreted, because the phenomena that occur in biological structures are stochastic and random. It is not possible to carry out a simulation for all available defined experimental values of parameters, which depend on many circumstances (age, occupation, gender, etc.). Therefore, investigators have decided to establish average values of these factors and use a deterministic model. To avoid this simplification and, at the same time, omit time-consuming stochastic calculation, it is sensible to use interval arithmetic. Results obtained using the interval arithmetic rules are represented in the form of ranges that contain correct simulation results. This is essential in biomechanics because thermophysical parameters, such as thermal conductivity or volumetric specific heat, can vary to some extent [38,39].

The paper presents the numerical analysis of the process of CPA transport across cell membrane during cryopreservation of articular cartilage samples. This model is weakly coupled to macroscale phenomena, such as heat and mass transfer, in the extracellular matrix. The LT protocol is used for the simulation. In the research, mass transport at the microscale is described by the interval 2-P formalism, which is complemented by results from the interval Fourier equation and the interval mass transfer equation based on the interval Fick’s second law. In the model, some thermophysical parameters are introduced using interval arithmetic, and, as a result, uncertain values will be properly construed. The interval results are compared with simulations data from Yu et al. [13].

## 2. Materials and Methods

The presented model simulates the cryopreservation process using the LT protocol proposed by Pegg et al. [5]. During the experiment, a cylindrical ovine articular cartilage sample is immersed in CPTes2 bathing solution, which is a potassium-rich mixture. In the investigations, the chemical composition of the CPA solution is simplified to DMSO, KCl, and H_2_O. Additionally, temperature and concentration of the mixture were controlled by a computer system. The state of the bathing solution refers to the boundary conditions, which supplemented the mathematical and numerical models.

## 2.1. Mathematical Model

The change in water volume in the chondrocytes and the change in the intracellular CPA mole number over time is simulated using an interval two-parameter model [13,14,40], which, for a cylindrical coordinate system, is of the form:

(1) $$\frac{d{\bar{V}}_{w}(r,z,t)}{dt}=-{\bar{L}}_{p}(r,z,t)AR_{g}\bar{T}(r,z,t)\left({\bar{M}}^{e}(r,z,t)-{\bar{M}}^{i}(r,z,t)\right),$$

(2) $$\frac{d{\bar{N}}_{d}(r,z,t)}{dt}={\bar{P}}_{s}(r,z,t)A\left({\bar{M}}^{e}(r,z,t)-{\bar{M}}^{i}(r,z,t)\right),$$

where ${\bar{V}}_{w}$(*r*, *z*, *t*) is the interval water volume in the chondrocyte, *t* is the time, *r* and *z* denote the cylindrical coordinates, ${\bar{N}}_{d}$ is the CPA mole number in the chondrocyte, ${\bar{L}}_{p}$(*r*, *z*, *t*) is the interval hydraulic conductivity, ${\bar{P}}_{s}$(*r*, *z*, *t*) is the interval permeability of CPA through the cell membrane, *A* is the chondrocyte surface area, *R_g_* is the gas constant equal to 8.314 J·mol^−1^·K^−1^, $\bar{T}$(*r*, *z*, *t*) is the interval tissue temperature, and $\bar{M}$(*r*, *z*, *t*) is the interval osmolarity, where the superscripts *e* and *i* refer to the extracellular and intracellular solutions, respectively.

The interval hydraulic conductivity, which determines the water permeability of a cell membrane, and the interval permeability of CPA across the cell membrane are described by the following equations [13]:

(3) $${\bar{L}}_{p}(r,z,t)=A_{Lp}\exp\left(\frac{E_{A,Lp}}{R_{g}\bar{T}(r,z,t)}\right),$$

(4) $${\bar{P}}_{s}(r,z,t)=A_{Ps}\exp\left(\frac{E_{A,Ps}}{R_{g}\bar{T}(r,z,t)}\right),$$

where *A_Lp_* and *A_Ps_* are pre-exponential factors, and *E_A_*_,*Lp*_ and *E_A_*_,*Ps*_ are activation energies for ${\bar{L}}_{p}$ and ${\bar{P}}_{s}$, respectively.

The osmolarity, which defines the number of moles of osmotically active substance in 1 L of solution, can be calculated on the basis of the osmolality of the solution. The osmolality is the number of moles of osmotically active substances dissolved in 1 kg of a solvent (e.g., water). For an undiluted solution of two solutes, the interval value of the osmolality can be expressed as [13,25]:

(5) $$\begin{array}{l} {\bar{\pi }}^{f}(r,z,t)=k_{diss}{\bar{m}}_{k}^{f}(r,z,t)+{\bar{m}}_{d}^{f}(r,z,t)+B_{k}{\left(k_{diss}{\bar{m}}_{k}^{f}(r,z,t)\right)}^{2}+\\ +B_{d}{\left({\bar{m}}_{d}^{f}(r,z,t)\right)}^{2}+(B_{k}+B_{d})k_{diss}{\bar{m}}_{k}^{f}(r,z,t){\bar{m}}_{d}^{f}(r,z,t) \end{array}$$

where ${\bar{\pi }}^{f}$ is the interval osmolality, *k_diss_* is the dissociation constant, ${\bar{m}}_{}^{f}$ is the interval molality, and *B* is the second osmotic virial coefficient, where the subscripts *k* and *d* represent the KCl and the CPA (exactly DMSO), respectively. Let us denote that the superscript *f* refers to the superscript *e* or *i* (extracellular or intracellular solution).

The molality of species *j* (e.g., *k* or *d*) is defined as the number of moles of the dissolved substance in 1 kg of solvent. When the interval mass fraction is known (the mass fraction is the ratio of the mass of a given component to the mass of the entire mixture), the interval molality can be determined:

(6) $${\bar{m}}_{j}^{f}(r,z,t)=\frac{{\bar{\omega }}_{j}^{f}(r,z,t)}{\left(1-{\bar{\omega }}_{j}^{f}(r,z,t)\right)M_{at.j}},$$

where ${\bar{\omega }}_{j}^{f}$ is the interval mass fraction of species *j* and *M_at.j_* is the molar mass of the species *j*. The molar mass corresponds to the molecular mass:

(7) $$M_{at.j}\approx M_{u,j}\cdot 1\left[g\cdot {\mathrm{mol}}^{-1}\right],$$

where *M_u_*_,*j*_ is the molecular mass of species *j*, which is given by the sum of the relative atomic mass of the elements.

The interval mass fraction can be calculated by the dependence [13]:

(8) $${\bar{\omega }}_{j}^{f}(r,z,t)={\bar{C}}_{j}^{f}(r,z,t)\frac{M_{at.j}}{\rho_{j}},$$

where ${\bar{C}}_{j}^{f}$ is the interval concentration of species *j* and ρ*_j_* is the density of species *j*. It should be noted that concentration is also called molarity and is defined as the mole number per liter of the solution.

After defining the interval osmolality (see Equation (5)), the interval osmolarity can be determined [13]:

(9) $${\bar{M}}^{f}(r,z,t)=\frac{{\bar{\pi }}^{f}(r,z,t){\bar{\omega }}_{w}^{f}(r,z,t)}{\sum {\bar{V}}_{j}^{f}(r,z,t)},$$

where ${\bar{V}}_{j}^{f}$ is the interval volume per unit of mass of species *j*. It should be noted that subscript *w* represents H_2_O.

The interval volume per unit mass is calculated [13]:

(10) $${\bar{V}}_{j}^{f}(r,z,t)=\frac{{\bar{\omega }}_{j}^{f}(r,z,t)}{M_{at.j}}v_{j},$$

where *v_j_* is the partial molar volume of species *j*.

It is also important to know the interval extracellular and intracellular concentrations, which are described by the following interval equations [13,14,15,18,22,27,40,41]:

(11) $$\frac{\partial {\bar{C}}_{d}^{e}(r,z,t)}{\partial t}=\left[\frac{1}{r}\frac{\partial }{\partial r}\left({\bar{D}}_{d}r\frac{\partial {\bar{C}}_{d}^{e}(r,z,t)}{\partial r}\right)+\frac{\partial }{\partial z}\left({\bar{D}}_{d}\frac{\partial {\bar{C}}_{d}^{e}(r,z,t)}{\partial z}\right)\right],$$

(12) $${\bar{C}}_{k}^{i}(r,z,t)=C_{k}^{e,0}\left(\frac{V_{cell}^{0}-{\bar{V}}_{b}(r,z,t)-v_{d}N_{d}^{0}}{{\bar{V}}_{cell}^{}(r,z,t)-{\bar{V}}_{b}(r,z,t)-v_{d}{\bar{N}}_{d}(r,z,t)}\right),$$

(13) $${\bar{C}}_{d}^{i}(r,z,t)=\frac{{\bar{N}}_{d}(r,z,t)}{{\bar{V}}_{cell}^{}(r,z,t)-{\bar{V}}_{b}(r,z,t)-v_{d}{\bar{N}}_{d}(r,z,t)},$$

where ${\bar{D}}_{d}$ is the interval diffusion coefficient, $V_{cell}^{0}$ is the cell volume (the superscript 0 represents the initial moment of simulation), ${\bar{V}}_{b}$ is the osmotically inactive volume of cells (for chondrocytes it is equal to 0.41 ${\bar{V}}_{0}$), where ${\bar{V}}_{0}$ is the isotonic volume of cells. The interval normalized cell volume is defined:

(14) $$\frac{{\bar{V}}_{cell}}{V_{cell}^{0}}=\frac{{\bar{V}}_{d}+{\bar{V}}_{w}}{V_{cell}^{0}}=\frac{{\bar{N}}_{d}v_{d}+{\bar{V}}_{w}}{V_{cell}^{0}},$$

The interval diffusion coefficient of CPA in extracellular matrix can be estimated by the Einstein–Stokes equation [41,42,43]:

(15) $${\bar{D}}_{d}(r,z,t)=\frac{k_{B}\bar{T}(r,z,t)}{6\pi \eta r_{s}},$$

where *k_B_* is the Boltzmann constant, equal to 1.38 × 10^−23^ J·K^−1^, *r_s_* is the radius of the spherical particle molecule, and η is the dynamic viscosity.

The interval temperature coupled to the interval mass transfer model (compare with Equations (1), (2) and (15)) is determined using the interval Fourier equation [13,41]:

(16) $$\bar{c}\frac{\partial \bar{T}(r,z,t)}{\partial t}=[\frac{1}{r}\frac{\partial }{\partial r}\left(\bar{\lambda }r\frac{\partial \bar{T}(r,z,t)}{\partial r}\right)+\frac{\partial }{\partial z}\left(\bar{\lambda }\frac{\partial \bar{T}(r,z,t)}{\partial z}\right)],$$

where $\bar{\lambda }$ is the interval thermal conductivity and $\bar{c}$ is the interval volumetric specific heat.

**Figure 1:**
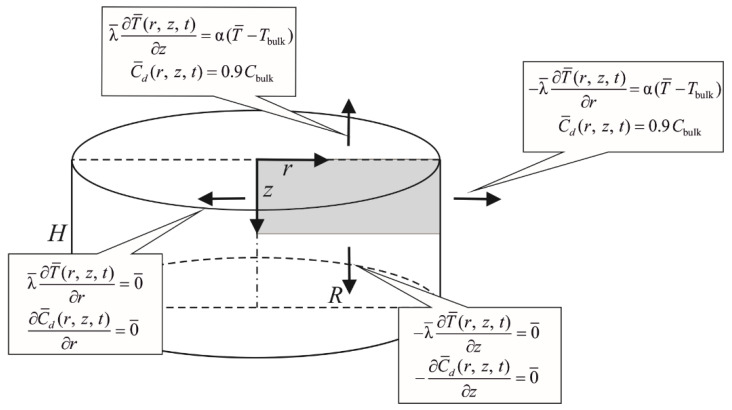
Domain considered and boundary conditions.

Equations (11)–(13) and (16) need to be supplemented with boundary conditions (see Figure 1) and initial conditions of the following form:

(17) $$t=0:\;\{\begin{array}{c} \bar{T}(r,z,0)=T^{0}\\ C_{d}^{e}(r,z,0)=C_{d}^{e,0}\\ C_{k}^{e}(r,z,0)=C_{k}^{e,0}\\ C_{k}^{i}(r,z,0)=C_{d}^{i}(r,z,0)=C_{}^{i,0} \end{array},$$

where $T_{\mathrm{bulk}}$ is the temperature of the bathing solution, $C_{\mathrm{bulk}}$ is the CPA concentration in the bathing solution, α is the natural convection heat transfer coefficient, $T^{0}$ is the initial temperature and $C_{d}^{e,0}$ the initial external DMSO concertation, $C_{k}^{e,0}$ is the initial external KCl concentration, and $C_{}^{i,0}$ is the initial internal DMSO and KCl concentration [13].

An axially symmetrical model of articular cartilage was considered, in particular the rectangle shown in Figure 1. Adiabatic boundary conditions were assumed on the symmetry axes (left and bottom sides), while on the other sides of the considered rectangle boundary conditions of 3rd type for temperature and 1st type for concentration were given.

The presented mathematical model does not include the phenomenon of phase changes. This is due to the fact that the liquidus tracking (LT) method is used to model heat and mass transfer. The LT protocol regulates the temperature and concentration in such a way that the temperature of the sample is above or on the liquidus line, which eliminates the probability of ice crystallization in cells—see the calculated eutectic temperatures and the melting points of tissue in [5].

## 2.2. Numerical Algorithm

Numerical model of thermal processes proceeding in domain of heating tissue is based on the finite difference method (FDM) in the version presented in [43,44].

**Figure 2:**
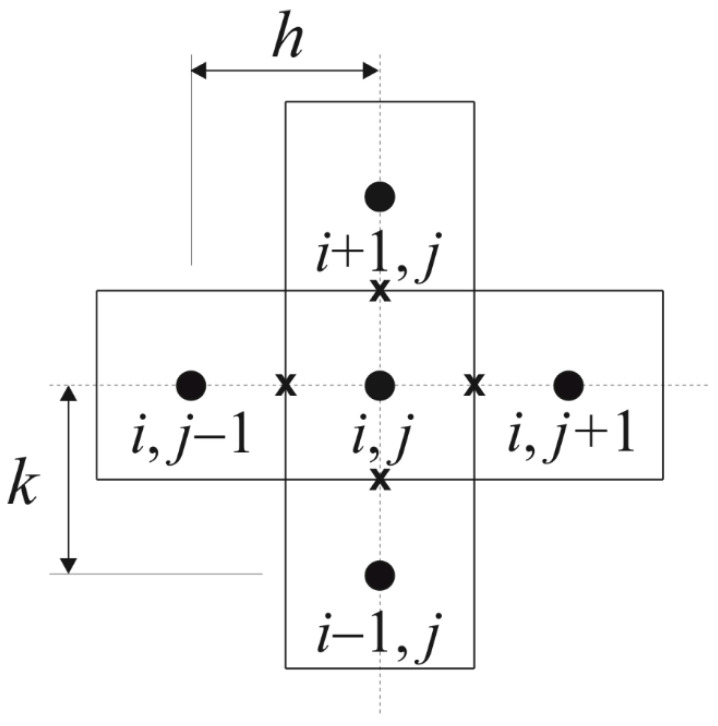
Five-point star.

A time grid with a constant step Δt and a geometrical mesh are introduced at the beginning. The boundary nodes are located at the distance 0.5 *h* or 0.5 *k* with respect to the real boundary *(h*, *k* are the steps of regular mesh in directions *r* and *z*), respectively (see Figure 2). This approach gives a better approximation of the Neumann and Robin boundary conditions [38,41,43].

The approximate form of the interval energy equation (Equation (16)) for the internal nodes (*i*, *j*) and transition *t^s−^*^1^ → *t^s^* is the following [41,43]:

(18) $$\begin{array}{c} {\bar{c}}_{i,j}^{s-1}\frac{{\bar{T}}_{i,j}^{s}-{\bar{T}}_{i,j}^{s-1}}{\Delta t}=\frac{\Phi_{i,j-1}^{}}{{\bar{R}}_{i,j-1}^{s-1}}\left({\bar{T}}_{i,j-1}^{s-1}-{\bar{T}}_{i,j}^{s-1}\right)+\frac{\Phi_{i,j+1}^{}}{{\bar{R}}_{i,j+1}^{s-1}}\left({\bar{T}}_{i,j+1}^{s-1}-{\bar{T}}_{i,j}^{s-1}\right)+\\ \frac{\Phi_{i-1,j}^{}}{{\bar{R}}_{i-1,j}^{s-1}}\left({\bar{T}}_{i-1,j}^{s-1}-{\bar{T}}_{i,j}^{s-1}\right)+\frac{\Phi_{i+1,j}^{}}{{\bar{R}}_{i+1,j}^{s-1}}\left({\bar{T}}_{i+1,j}^{s-1}-{\bar{T}}_{i,j}^{s-1}\right) \end{array},$$

where

(19) $$\begin{array}{ccc} \Phi_{i,j-1}^{}=\frac{r_{i,j}-0.5h}{r_{i,j}h}, & \Phi_{i,j+1}^{}=\frac{r_{i,j}+0.5h}{r_{i,j}h}, & \Phi_{i-1,j}^{}=\Phi_{i+1,j}^{}=\frac{1}{k} \end{array},$$

are the shape functions of differential mesh, while the thermal resistances are defined by the following formulas:

(20) $$\begin{array}{cc} {\bar{R}}_{i,j+1}^{s-1}=\frac{0.5h}{{\bar{\lambda }}_{i,j}^{s-1}}+\frac{0.5h}{{\bar{\lambda }}_{i,j+1}^{s-1}}, & {\bar{R}}_{i,j-1}^{s-1}=\frac{0.5h}{{\bar{\lambda }}_{i,j}^{s-1}}+\frac{0.5h}{{\bar{\lambda }}_{i,j-1}^{s-1}} \end{array},$$

(21) $$\begin{array}{cc} {\bar{R}}_{i+1,j}^{s-1}=\frac{0.5k}{{\bar{\lambda }}_{i,j}^{s-1}}+\frac{0.5k}{{\bar{\lambda }}_{i+1,j}^{s-1}}, & {\bar{R}}_{i-1,j}^{s-1}=\frac{0.5k}{{\bar{\lambda }}_{i,j}^{s-1}}+\frac{0.5k}{{\bar{\lambda }}_{i-1,j}^{s-1}} \end{array}.,$$

The interval mass diffusion equation (Equation (11)) is transformed in a similar way

(22) $$\begin{array}{c} \frac{{\left({\bar{C}}_{d}^{e}\right)}_{i,j}^{s}-{\left({\bar{C}}_{d}^{e}\right)}_{i,j}^{s-1}}{\Delta t}=\frac{\Phi_{i,j-1}^{}}{{\bar{W}}_{i,j-1}^{s-1}}\left[{\left({\bar{C}}_{d}^{e}\right)}_{i,j-1}^{s-1}-{\left({\bar{C}}_{d}^{e}\right)}_{i,j}^{s-1}\right]+\frac{\Phi_{i,j+1}^{}}{{\bar{W}}_{i,j+1}^{s-1}}\left[{\left({\bar{C}}_{d}^{e}\right)}_{i,j+1}^{s-1}-{\left({\bar{C}}_{d}^{e}\right)}_{i,j}^{s-1}\right]\\ +\frac{\Phi_{i-1,j}^{}}{{\bar{W}}_{i-1,j}^{s-1}}\left[{\left({\bar{C}}_{d}^{e}\right)}_{i-1,j}^{s-1}-{\left({\bar{C}}_{d}^{e}\right)}_{i,j}^{s-1}\right]+\frac{\Phi_{i+1,j}^{}}{{\bar{W}}_{i+1,j}^{s-1}}\left[{\left({\bar{C}}_{d}^{e}\right)}_{i+1,j}^{s-1}-{\left({\bar{C}}_{d}^{e}\right)}_{i,j}^{s-1}\right] \end{array},$$

where the diffusion resistances between the central node and the adjoining ones are the following:

(23) $$\begin{array}{cc} {\bar{W}}_{i,j+1}^{s-1}=\frac{0.5h}{{\left({\bar{D}}_{d}\right)}_{i,j}^{s-1}}+\frac{0.5h}{{\left({\bar{D}}_{d}\right)}_{i,j+1}^{s-1}}, & {\bar{W}}_{i,j-1}^{s-1}=\frac{0.5h}{{\left({\bar{D}}_{d}\right)}_{i,j}^{s-1}}+\frac{0.5h}{{\left({\bar{D}}_{d}\right)}_{i,j-1}^{s-1}} \end{array},$$

(24) $$\begin{array}{cc} {\bar{W}}_{i+1,j}^{s-1}=\frac{0.5k}{{\left({\bar{D}}_{d}\right)}_{i,j}^{s-1}}+\frac{0.5k}{{\left({\bar{D}}_{d}\right)}_{i+1,j}^{s-1}}, & {\bar{W}}_{i-1,j}^{s-1}=\frac{0.5k}{{\left({\bar{D}}_{d}\right)}_{i,j}^{s-1}}+\frac{0.5k}{{\left({\bar{D}}_{d}\right)}_{i-1,j}^{s-1}} \end{array},$$

Details of the above transformations are presented in [41].

The final form of the interval FDM equations (Equations (1) and (2)) of the two-parameter model for the internal nodes are

(25) $${\bar{V}}_{w\;i,j}^{s}={\bar{V}}_{w\;i,j}^{s-1}+\Delta t{\left[-{\bar{L}}_{p}AR_{g}\bar{T}\left({\bar{M}}^{e}-{\bar{M}}^{i}\right)\right]}_{i,j}^{s-1},$$

(26) $$N_{d\;i,j}^{s}=N_{d\;i,j}^{s-1}+\Delta t{\left[{\bar{P}}_{s}A\left({\bar{M}}^{e}-{\bar{M}}^{i}\right)\right]}_{i,j}^{s-1},$$

The systems of Equations (18), (22), (25), and (26) have been solved using the assumption of the stability condition for the explicit differential scheme [43,44].

All mathematical operations leading to the determination of the temperatures, extracellular and intracellular concentrations and other sought quantities require the application of directed arithmetic rules—more information can be found in [41].

## 3. Results

The simulation of CPA transport across a cell membrane for the homogenous cylindrical articular cartilage sample was carried out. The dimensions of the sample were as follows: *H* = 1 mm and *R* = 3 mm. The thermophysical parameters of the tissue and the chemical properties of CPA, including DMSO, KCl and H_2_O, are given in Table 1. The chondrocytes’ surface area and the chondrocytes’ isotonic radius have been introduced, where *A* = 8.04 × 10^−10^ m^2^ and *r_cell_* = 8 × 10^−6^ m, which allows to calculate the isotonic volume of the cells $V_{0}^{}$ = 2.14 × 10^−15^ m^3^. It is assumed that the initial cell volume is equal to the isotonic volume of the cells $V_{cell}^{0}=V_{0}^{}$. Additionally, the following input data were used: initial temperature *T*^0^ = 22 °C, initial external DMSO concentration $C_{d}^{e,0}$ = 0% (*w/w*), initial external KCl concentration $C_{k}^{e,0}$ = 0.85% (*w/w*), initial internal DMSO and KCl concentrations $C_{}^{i,0}$ = 0% (*w/w*), pre-expositional factors, such as *A_Lp_ =* 9.1 × 10^−6^ m^2^·s·kg^−1^ and *A_Ps_ =* 1.2 × 10^12^ m·s^−1^ and the activation energy, *E_A_*_,*Lp*_ = 45.73 × 10^3^ J mol^−1^ and *E_A_*_,*Ps*_ = 107.40 × 10^3^ J mol^−1^ [13,41].

The process was simulated using the interval finite difference method. The time step and the mesh steps were assumed as Δ*t* = 0.001 s, *h* = 0.0001 m and *k* = 0.00005 m.

**Figure 3:**
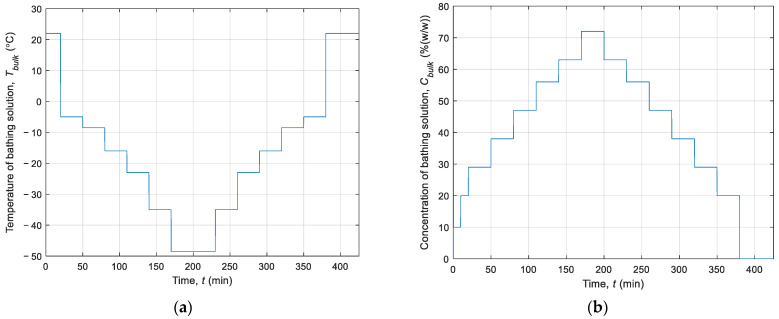
History of (**a**) the temperature and (**b**) the concentration of the bathing solution.

During modelling of the cryopreservation process, the LT protocol introduced by Pegg et al. in [5] was used. The procedure includes 8 steps in a cooling phase and 7 steps in a heating phase. During each step, the temperature and concentration of the bathing solution were controlled by computer. This ensured that no ice crystals were formed and that no cells were damaged by toxicity. It is worth mentioning that the impact of CPA causes a change in the melting point, and then the temperature of the cryopreserved sample was close to the liquidus track. Table 2 and Figure 3 present the assumption of the LT approach. The given values of the temperature and concentration of the bathing solution corresponded to the boundary conditions of the mathematical model.

**Figure 4:**
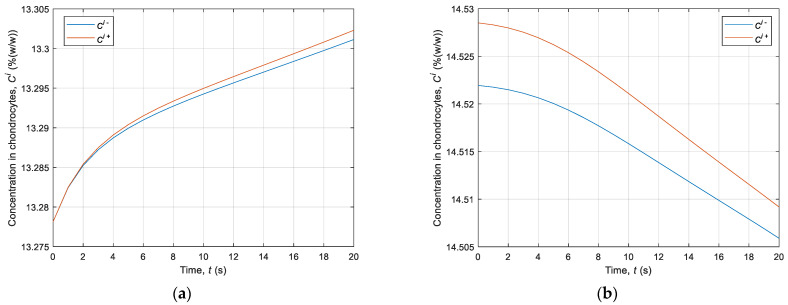
History of the intracellular concentration over time: (**a**) from 22 °C to −5 °C; (**b**) from −5 °C to 22 °C.

**Figure 5:**
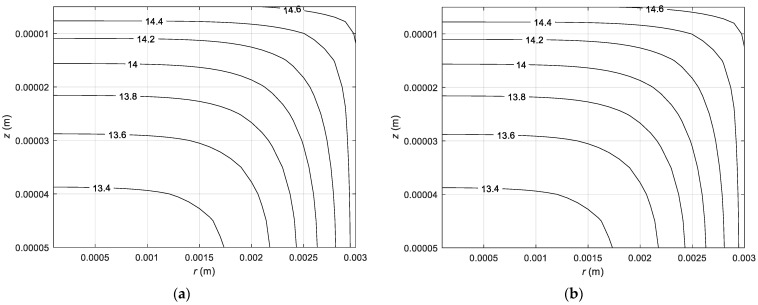
Distribution of the intracellular concentration after 10 s for transition from 22 °C to −5 °C for: (**a**) $C_{d}^{i}{}^{-}$; (**b**) $C_{d}^{i}{}^{+}$.

Firstly, simulation of the CPA concentration in the intracellular membrane using directed interval arithmetic was executed. Figure 4 illustrates the distribution of the concentration in chondrocytes located at the point with the coordinates *r* = 0.1 mm and *z* = 0.45 mm. This refers to the first 20 s in step 3 of the cooling phase (Figure 4a) and step 7 of heating phase (Figure 4b). It should be pointed out that, for each node of the domain considered, there are two curves representing the beginning and end of concentration intervals. The upper limit of the concentration interval is marked with a red line and the lower limit with a blue line. Figure 5 represents the CPA concentration in the entire analyzed area after 10 s in step 3 in the cooling phase.

**Figure 6:**
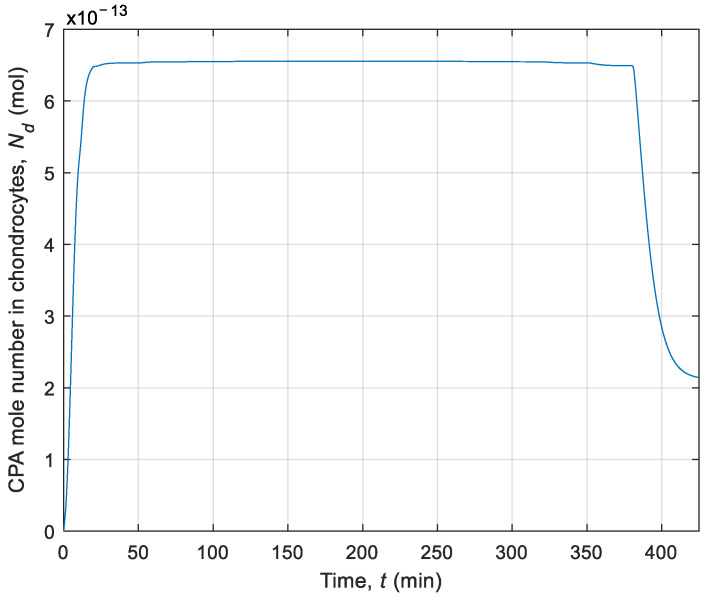
History of CPA mole number in chondrocytes during cryopreservation.

**Figure 7:**
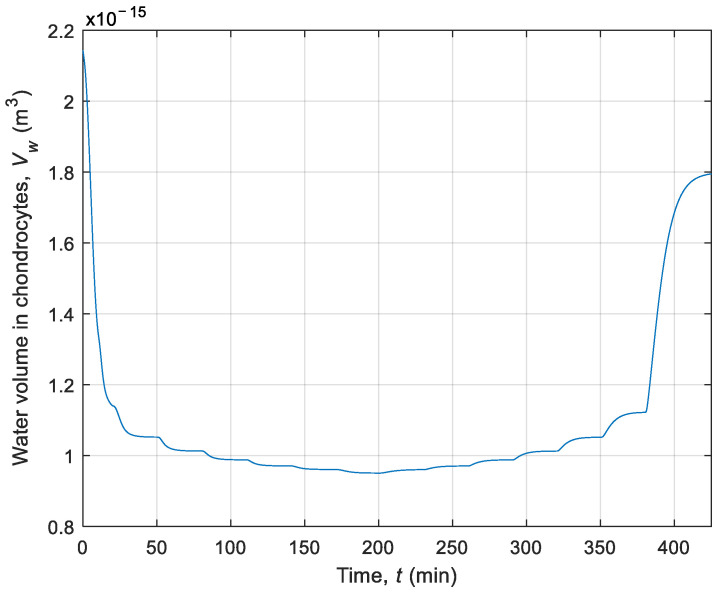
History of water volume in chondrocytes during cryopreservation.

Afterwards, to better understand the phenomena occurring in chondrocytes, the simulation of CPA and water volume changing in the cell was performed without using interval arithmetic. In this model, the thermophysical parameters were defined as deterministic values (see deterministic values of λ and *c* in Table 1). Figure 6 shows a diagram of the number of moles of CPA over time during the whole process. Figure 7 presents changes in the intracellular water volume during cryopreservation. This diagram expresses the phenomenon of dehydration, which consists of water transport from the inside cell to the extracellular matrix.

**Figure 8:**
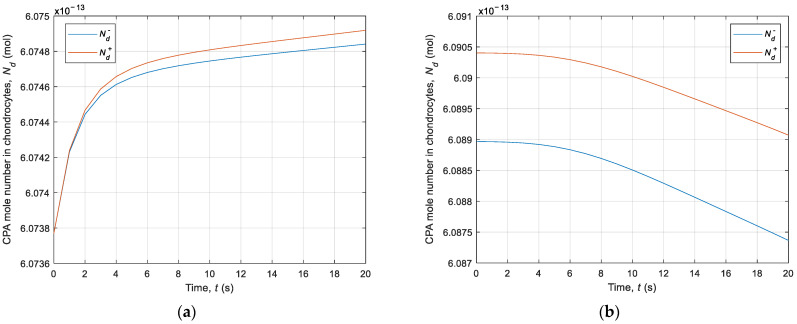
History of the intracellular CPA moles number over time: (**a**) from 22 °C to −5 °C; (**b**) from −5 °C to 22 °C.

**Figure 9:**
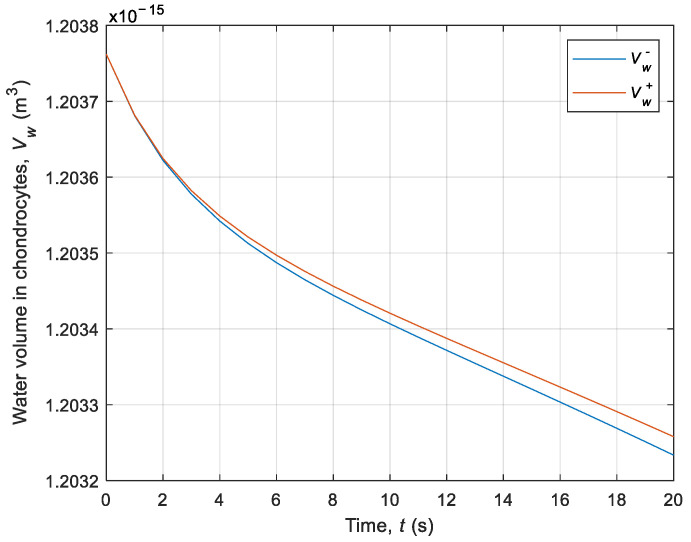
History of the intracellular water volume over time for transition from 22 °C to −5 °C.

Then, the intracellular volume of water and CPA was also analyzed using interval arithmetic rules. Figure 8 demonstrates the CPA moles’ number in the cell for the first 20 s in step 3 of the cooling phase and step 7 of the heating phase. These diagrams correspond to the intracellular CPA concertation (compare with Figure 4). Figure 9 shows a diagram of water volume over time for transition from 22 °C to −5 °C. It should be noted that the obtained intervals are narrow.

Table 3 includes the values of intracellular quantities at the end of the given steps. The exact results of the extracellular temperature and CPA concentration can be found in previous work [41]. The obtained interval concentrations were also compared with the simulation data received by Yu et al. [13]. In individual steps, these values are close to each other. Significant discrepancies occur for steps where the temperature is low.

## 4. Discussion

In the present study, numerical analysis of heat and mass transfer occurring in axially symmetrical articular cartilage sample subjected to the cryopreservation process is presented. The mathematical model is based on heat and mass diffusion interval equations, in conjunction with a model of transmembrane transport of CPA. A two-parameter model was used to analyze the transport of CPA (solution of DMSO, KCl and water) across the cell membrane, considering the simulated volume of water in chondrocytes and the change in the cryoprotectant concentration over time. Interval values of volume specific heat and thermal conductivity of articular cartilage were assumed for the modelled process. The interval FDM was used to solve the problem, applying the rules of directed interval arithmetic.

Since thermophysical parameters are often determined experimentally, as well as the phenomena that biological structures are unpredictable, it is reasonable to include in the mathematical model imprecise values instead of deterministic values. The application of the stochastic and randomized model is time-consuming and complicated, therefore applying interval arithmetic rules is proposed in the research. Using interval values, solutions in the form of intervals were obtained, which better reflect the analyzed process.

In the paper, the history of the intracellular concentration, the history of the intracellular water volume in the chondrocytes and the history of the intracellular CPA moles number in the cell have been presented. The results depict the phenomena that occur in cells during the process, such as dehydration. It can be also seen that, for low temperatures, wider intervals were obtained, while for positive temperatures the intervals were narrow. The obtained interval concentrations were compared with simulation data calculated using a deterministic model [13]. The interval results mostly coincide with Yu et al.’s simulation data. Slight differences are observed in those process steps in which the concentration is high (e.g., step 8 in the cooling phase or step 1 in the heating phase). The comparison of the deterministic model with the interval model allows us to conclude that the model modified by interval arithmetic is justified because the results are compatible, and the received ranges include the correct results, whereas the computation time is only slightly extended.

In a further stage of the study, the macroscale mass transfer equation will be supplemented with a velocity vector determined from the Navier–Stokes equation.

## Figures and Tables

**Table 1:** Thermophysical parameters of the tissue and chemical properties of the CPA [13,41,45].

Thermophysical Tissue Parameters				
Parameter		Value		
$\bar{\lambda }$	(W·m^−1^·K^−1^)	$\bar{\lambda }=\left[\lambda -0.05\lambda ,\lambda +0.05\lambda \right]$, where λ = 0.518		
$\bar{c}$	(J·m^−3^·K^−1^)	$\bar{c}=\left[c-0.05c,c+0.05c\right]$, where *c* = 3.924 × 10^6^		
α	(W·m^−2^·K^−1^)	525		
**CPA Chemical Properties**				
Parameter		Values		
		DMSO	KCl	H_2_O
*r_s_*	(m)	2.541 × 10^−10^	-	-
η	(Pa·s)	1.996 × 10^−3^	-	-
ρ	(kg·m^−3^)	1.1 × 10^3^	1.98 × 10^3^	997
*M_at._*	(kg·mol^−1^)	78.13 × 10^−3^	74.5513 × 10^−3^	18.015 × 10^−3^
*B*	(kg·mol^−1^)	0.108	0	-
*v*	(L·mol^−1^)	70.97 × 10^−3^	37.5 × 10^−3^	18.07 × 10^−3^
*k_diss_*	-	-	1.772	-

**Table 2:** The assumption of the LT protocol [5,13].

Phase	Step	Time	Temperature of Bathing Solution	Concentration of Bathing Solution
		*t* (min)	*T_bulk_* (°C)	*C_bulk_* (%(*w/w*))
Cooling	1	10	22	10
	2	10	22	20
	3	30	−5	29
	4	30	−8.5	38
	5	30	−16	47
	6	30	−23	56
	7	30	−35	63
	8	30	−48.5	72
Heating	1	30	−48.5	63
	2	30	−35	56
	3	30	−23	47
	4	30	−16	38
	5	30	−8.5	29
	6	30	−5	20
	7	45	22	0

**Table 3:** Values of intracellular quantities at the end of each step.

**Phase**	**Step**	CPA Concentration		Yu et al.’s Simulation Data [13]	CPA Volume		CPA Moles Number		Water Volume	
		$C_{d}^{i}{}^{-}(\%(ww))$	$C_{d}^{i}{}^{+}(\%(ww))$	*C_d_* (%(*w/w*))	$V_{d}^{}{}^{-}(m^{3})\;\times \;10^{-17}$	$V_{d}^{}{}^{+}(m^{3})\;\times \;10^{-17}$	$N_{d}^{}{}^{-}(\mathrm{mol})\;\times \;10^{-13}$	$N_{d}^{}{}^{+}(\mathrm{mol})\;\times \;10^{-13}$	$V_{w}^{}{}^{-}(m^{3})\;\times \;10^{-16}$	$V_{w}^{}{}^{+}(m^{3})\;\times \;10^{-16}$
Cooling	1	4.330	4.330	6.70	2.816	2.816	3.967	3.967	15.297	15.297
	2	13.278	13.278	15.68	4.311	4.311	6.074	6.074	12.038	12.038
	3	21.513	21.520	24.42	4.358	4.362	6.140	6.147	10.816	10.818
	4	29.172	29.182	31.86	4.371	4.379	6.160	6.170	10.288	10.290
	5	37.371	37.385	38.33	4.375	4.383	6.164	6.176	9.960	9.962
	6	46.059	46.076	44.02	4.376	4.385	6.166	6.179	9.740	9.741
	7	53.079	53.107	47.26	4.376	4.385	6.166	6.179	9.614	9.615
	8	62.071	62.338	48.63	4.376	4.385	6.166	6.179	9.494	9.493
Heating	1	53.272	53.921	50.32	4.376	4.385	6.166	6.179	9.600	9.613
	2	46.155	46.185	50.72	4.376	4.385	6.166	6.179	9.737	9.740
	3	37.452	37.453	44.99	4.375	4.383	6.164	6.176	9.958	9.960
	4	29.238	29.236	36.27	4.371	4.379	6.160	6.170	10.285	10.287
	5	21.575	21.573	27.05	4.358	4.363	6.140	6.147	10.810	10.812
	6	14.522	14.529	18.86	4.321	4.3212	6.089	6.090	11.765	11.768
	7	0.033	0.033	0.03	0.040	0.041	0.057	0.058	21.100	21.103

## Data Availability

Data sharing not applicable.
